# Modifiable Risk Factors for Mild Cognitive Impairment in Elderly Populations: Insights from a Shanghai Community Cohort Study

**DOI:** 10.1002/alz70860_100056

**Published:** 2025-12-23

**Authors:** Ling Yue, Xinyi Qian, Bo Hong, Yuanyuan Liu, Ning Su, Wei Li, Yewei Wang, Xia Li, Shifu Xiao

**Affiliations:** ^1^ Shanghai Mental Health Center, Shanghai Jiao Tong University School of Medicine, Shanghai, China; ^2^ Alzheimer's Disease and Related Disorders Center, Shanghai Jiao Tong University, Shanghai, Shanghai, China; ^3^ Alzheimer's Disease and Related Disorders Center, Shanghai Jiao Tong University, Shanghai, China

## Abstract

**Background:**

Mild cognitive impairment (MCI) is a critical stage in dementia prevention. This study aims to explore key modifiable risk factors for MCI among community‐dwelling elderly in Shanghai, China, by incorporating potential factors unique to China into the established list of modifiable risk factors at an earlier stage.

**Method:**

1000 participants with a baseline diagnosis of normal cognition (NC) were selected and involved in this study through the Shanghai Brain Health Cohort Study. All the participants completed a self‐administered questionnaire collecting demographic, lifestyle, and clinical information, as well as a comprehensive neuropsychological assessment at both baseline and each two‐year follow‐up visit. For continuous variables among the 7 categories of modifiable risk factors that were statistically significant in the univariate COX regression analysis, the Maximally Selected Log‐Rank Statistic was used to determine the optimal cutoff point based on the KM curve, converting them into binary variables for Population Attributable Fraction (PAF) calculation.

**Result:**

Participants had a mean age of 68.31±7.07 years, with 65.5% being female. During the follow‐up period, 267 individuals (26.7%) developed MCI. Seven modifiable risk factors were identified: being a manual laborer (HR = 1.02, *p* = 0.042), abnormal marital status (divorced or widowed) (HR = 1.49, *p* = 0.016), comorbidity including diabetes (HR = 1.73, *p* < 0.001) and cerebral infarction (HR = 1.51, *p* = 0.007), depression (HR = 1.03, *p* = 0.03), chronic sleep disorder (HR = 1.03, *p* = 0.03), and abnormal sleep duration in both youth (HR = 0.89, *p* = 0.026) and middle age (HR = 0.87, *p* = 0.008). Additionally, eating soy products was found to be protective (HR = 0.75, *p* = 0.031). The overall weighted population attributable fraction (PAF) for these risk factors was 24.17%, which increased to 24.39% after adjusting for age and years of education.

**Conclusion:**

These findings suggest that comorbidity with diabetes and cerebral infarction, manual labor status, abnormal sleep duration, and not consuming soy products are major modifiable risk factors for MCI in the study population, offering novel insights and potential new research directions for the early prevention of dementia.

